# Indirect Thermographic Measurement of the Temperature of a Transistor Die during Pulse Operation

**DOI:** 10.3390/s24196452

**Published:** 2024-10-06

**Authors:** Arkadiusz Hulewicz, Krzysztof Dziarski, Łukasz Drużyński

**Affiliations:** 1 Institute of Electrical Engineering and Electronics, Poznan University of Technology, Piotrowo 3A, 60-965 Poznan, Poland; 2 Institute of Electric Power Engineering, Poznan University of Technology, Piotrowo 3A, 60-965 Poznan, Poland; krzysztof.dziarski@put.poznan.pl (K.D.); lukasz.druzynski@put.poznan.pl (Ł.D.)

**Keywords:** thermography, C2M0280120D, transistor, switching, thermal modelling, solidworks

## Abstract

This paper presents aspects related to the indirect thermographic measurement of a C2M0280120D transistor in pulse mode. The tested transistor was made on the basis of silicon carbide and is commonly used in many applications. During the research, the pulse frequency was varied from 1 kHz to 800 kHz. The transistor case temperature was measured using a Flir E50 thermographic camera and a Pt1000 sensor. The transistor die temperature was determined based on the voltage drop on the body diode and the known characteristics between the voltage drop on the diode and the temperature of the die. The research was carried out in accordance with the presented measuring standards and maintaining the described conditions. The differences between the transistor case temperature and the transistor die temperature were also determined based on simulation work performed in Solidworks 2020 SP05. For this purpose, a three-dimensional model of the C2M0280120D transistor was created and the materials used in this model were selected; the methodology for selecting the model parameters is discussed. The largest recorded difference between the case temperature and the junction temperature was 27.3 °C. The use of a thermographic camera allows the transistor’s temperature to be determined without the risk of electric shock. As a result, it will be possible to control the C2M0280120D transistor in such a way so as not to damage it and to optimally select its operating point.

## 1. Introduction

Transistors are among the electronic components from which electronic devices are built [1]. They consist of a die placed on a base plate made of a well-conducting metal, which, in many cases, is copper. The die is encapsulated inside epoxy resin, and connections to die are possible using leads, commonly called “legs”, which are often made of the same material as the base plate. Only one of the leads is directly attached to the base plate. The remaining two leads are placed in the epoxy resin and connected to the die using thin bond wires. The parts of the base plate and leads that are not placed under the epoxy resin layer are covered with a thin layer of tin [2,3]. The dimensions of the base plate, leads, and the epoxy resin layer depend on the type of case used; the most commonly used cases are TO 220 and TO 247. The dimensions of the electronic cases are standardized [4]. In the remainder of this article, the term “transistor” should be understood together with the case in which it is placed.

The most popular materials from which transistor dies are made include silicon (Si), silicon carbide (SiC), and gallium nitride (GaN). Transistors made of these materials differ in their properties [5]. Electronic components made on the basis of silicon reach the limit operating parameters resulting from the theoretical limitations of the material used. For this reason, electronic components based on SiC and GaN materials, called wide band gap (WBG) semiconductors, are becoming more and more popular. They feature better electrical, mechanical, and thermal properties than the electronic components made on the basis of Si. Replacing Si with WBG semiconductors increases the breakdown voltages, operating temperatures, and switching frequency and reduces switching losses [6]. Elements made on the basis of SiC deserve special attention. Compared to Si elements, those made on the basis of SiC have higher breakdown voltage values and higher thermal conductivity [7]. Another feature of this type of semiconductor is low ON-resistance [8].

Metal oxide semiconductor field effect transistors (MOSFETs) made on the basis of SiC are used in the construction of high conversion ratio converters (HCRCs) [9], traction converters [10], wind turbine converters [11], motor drives for electric vehicles [12], and DC–DC step-up converters [13]. The operational reliability of these devices is related to the operational reliability of the SiC MOSFETs placed inside them [14]. Due to the construction of the die area and the width of the gate oxide, they are susceptible to transient-overloading or short-circuit events [15]. Other examples of SiC MOSFET damage are related to long-term exposure to high temperature, which can cause interlayer dielectric erosion, electrode delamination, gate-oxide breakdown [16], and bond-wire lift-off and solder cracks [17,18].

The temperature value of SiC MOSFETs depends, among other things, on their switching frequency [19]. In turn, the switching frequency depends on the operating characteristics of the device in which the transistor is placed and on its energy efficiency [20]. A good example is the converter. In high-power converters, lower switching frequencies are often used to minimize switching losses and increase energy efficiency [21]. In turn, in low-power converters, higher switching frequencies are usually used, which may lead to smaller converter sizes and better regulation [22]. The higher the switching frequency is, the shorter is the switching time of SiC-based MOSFETs. During switching, rapid changes in voltage and current occur, which leads to power losses and heat generation. Therefore, the switching frequency has a direct impact on heat generation in the transistor [23].

The switching frequency of a SiC MOSFET affects the temperature of its die. In turn, the operation of the die at excessively high temperature may damage the transistor. For this reason, it is necessary to monitor the die temperature of the transistor, *T_j_*. In the literature, is possible to find three groups of methods that make this possible: electrical, contact, and non-contact methods [24].

Electrical methods use a selected parameter whose value depends on *T_j_*. This parameter is called the temperature-sensitive parameter (TSP) [25]. An example of a TSP that is used to determine the die temperature of a transistor is the drop voltage across the body diode. Knowing the relationship between TSP and *T_j_*, it is possible to determine the *T_j_* value based on the measured TSP value. The relationship between TSP and *T_j_* is individual for each transistor. Additionally, its determination requires removing the transistor from the device in which it was installed and placing it in the measurement system. For this reason, this method is not suitable for real-time monitoring of *T_j_* values [26].

Contact methods involve applying a temperature sensor to the transistor package (also called a ‘case’ in the literature) or directly to the die transistor. There is thermal resistance of an unknown value between the temperature sensor case and the transistor case (or die). Additionally, touching the transistor (or die) case with the temperature sensor causes a local disturbance of the temperature distribution. Part of the transistor case is made of metal; therefore, incorrect application of the temperature sensor (especially when placed in a metal case) may cause electric shock [27].

Non-contact methods are based on the absorption of infrared radiation emitted from the surface of the transistor case (indirect non-contact method) or through the transistor die (direct non-contact method). One of these methods is infrared thermography, which is considered safe, as it poses no risk of electric shock (e.g., as a result of touching a metal temperature sensor based on a plate or a radiator to which a transistor is attached). The direct method requires opening the transistor case. It is difficult to close the opened case. For this reason, it is not suitable for real-time application. The use of the indirect non-contact method consists of two steps: measuring the temperature of the transistor case (*T_c_*) and determining the difference between *T_c_* and *T_j_. T_c_* can be measured using a pyrometer and a thermographic camera. The use of a thermographic camera makes it possible to determine the temperature distribution on the surface of the transistor case. The differences between *T_c_* and *T_j_* can be determined using the finite element method. Knowing the value of the thermographic measurement of the temperature of the transistor case and the relationship between *T_c_* and *T_j_*, it is possible to determine the value of *T_j_* in real time [28,29].

After analyzing the available sources, no studies were found on the indirect thermographic temperature measurement of a SiC MOSFET, the temperature of which increases due to the increase in the switching frequency. For this reason, it was decided to undertake research that would result in the development of a method enabling the indirect thermographic measurement of the SiC MOSFET die temperature and monitoring that temperature at variable switching frequencies.

Section 2 describes the tested SiC MOSFET, the methodology, and the measurement system; Section 3 describes the obtained results of the work; Section 4 contains a discussion; and Section 5 presents conclusions.

## 2. Tested Transistor, Methodology, and Measurement System

**Figure 1:**
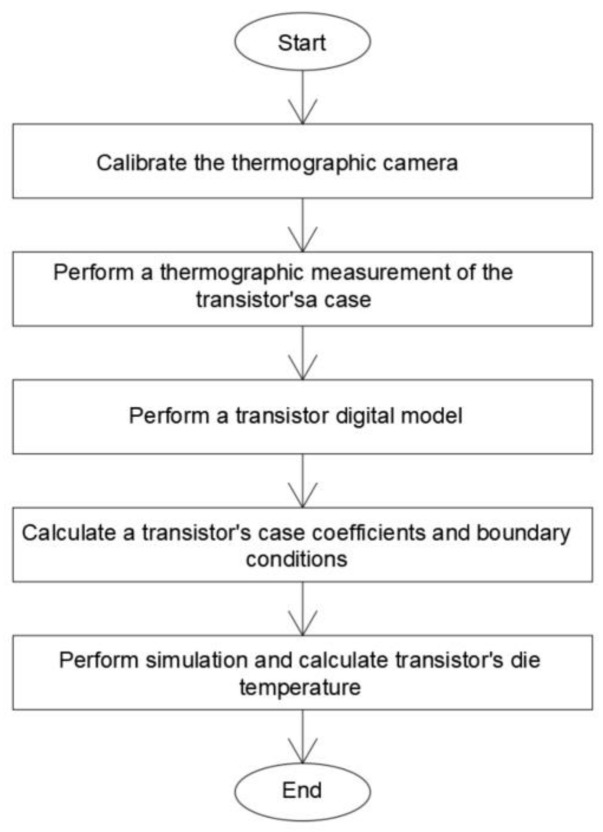
Algorithm for performing indirect thermographic temperature measurements of a transistor.

The indirect thermographic measurement of the transistor temperature die consists of two parts. The first part consists of performing a thermographic measurement of the transistor case temperature, *T_c_*. The second part consists of determining the transistor die value *T_j_* using simulation work. The method of performing a thermographic measurement of *T_c_* is described in Section 2.1. The value of *T*_Pt1000_, which is used to verify the *T_c_* value, is also determined and described in Section 2.1. The method used to determine the *T_j_* value based on simulation work is described in Section 2.2. The method for determining the *T_jd_* value, which is used to verify the *T_j_* value (determined based on simulation work), is also described in Section 2.2. The algorithm for the procedure is presented in Figure 1.

### 2.1. Tested Transistor and Measurement System

**Figure 2:**
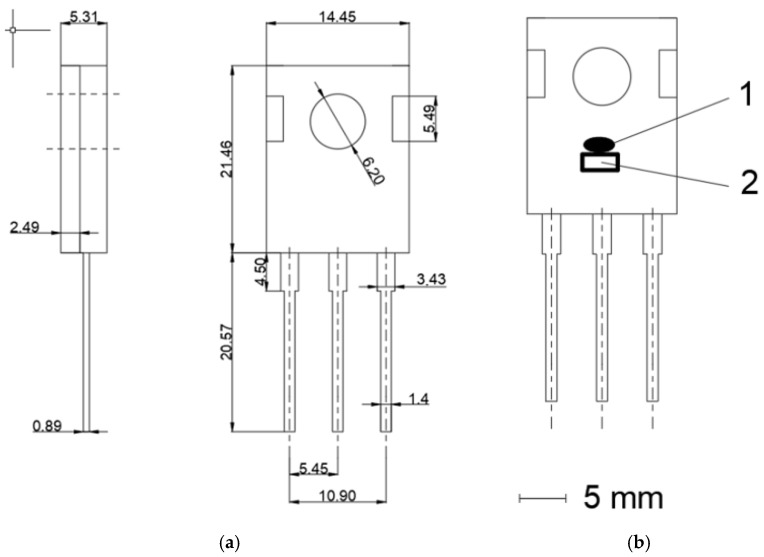
(**a**) External dimensions of transistor model C2M0280120D in a TO 247 case. (**b**) Schematic of C2M0280120D in a TO 247 case. A marker was painted on the case using Velvet Coating 811-21 paint (1) and a Pt1000 sensor was glued onto the case (2).

The model C2M0280120D (Cree Inc., Durham, NC, USA) transistor was selected for testing. This transistor is described by the following parameters: *V_DSmax_* = 1200 V (for *V_GS_* = 0 V, *I_D_* = 100 µA), *V_GSmax_* = −10/+25 V, *I_D_* = 10 A (for *V_GS_* = 20 V, *T_c_* = 25 °C), *I_D_* = 6 A (for *V_GS_* = 20 V, *T_c_* = 100 °C), and *I_Dpulse_* = 20A. The external dimensions of the transistor and schematics are shown in Figure 2. Three randomly selected C2M0280120D transistors from the same series were selected to carry out the work.

Pt1000 sensors in an SMD 6203 case (Reichelt electronics GmbH & Co. KG, Sande, Germany) were glued to the case of each transistor [30]. For this purpose, WLK 5 glue with a known thermal conductivity value of *k* = 0.836 W/mK (Fischer Elektronik GmbH & Co. KG, Lüdenscheid, Germany) was used [31]. Additionally, next to the Pt1000 sensor, a measurement marker was painted on the transistor case. Velvet Coating 811-21 (Nextel, Hamburg, Germany) paint was used for this purpose with a known emissivity coefficient value *ε* ranging from 0.970 to 0.975 for temperatures within the range from –36 °C to 82 °C. The uncertainty with which the emissivity coefficient value was determined was 0.004 [32].

The transistor prepared in this way was placed in a station where the measuring device was a Flir E50 Thermographic Camera (Flir, Wilsonville, OR, USA) [33]. The selected Flir E50 thermographic camera was equipped with a matrix from an uncooled IR detector (7.5–13 µm) with a resolution of 240 × 180 pixels and an instantaneous field of view (IFOV) value of 1.82 mrad. The noise equivalent differential temperature (NEDT) value of this camera was 50 mK. An additional Close-up 2× lens (T197214, Flir, Wilsonville, OR, USA) was attached to the camera lens [34]. As a consequence, it was possible to obtain an IFOV value of 67 µm for the above-mentioned detector array (240 × 180 pixels) (thermographic camera with the additional lens). Before starting the work, the correctness of the indications of the camera used was verified using the IRS Calilux thermographic camera calibration standard (AT—Automation Technology GmbH, Bad Oldesloe, Germany) [35].

The thermographic camera prepared in this way was placed together with the tested C2M0280120D transistor in a chamber made of plexiglass. The external dimensions of the chamber were 45 cm × 35 cm × 35 cm. The internal dimensions of the chamber were 40 cm × 30 cm × 30 cm. The difference resulted from two reasons: the thickness of the plexiglass used (3 mm) and the thickness of the material (black foam made of polyurethane) lining the internal walls of the chamber. The foam used is characterized by porous structure, and every single pore of the foam resembles the black body cavity model. As a consequence, the material used was characterized by having a high emissivity factor *ε* = 0.95 [36].

**Figure 3:**
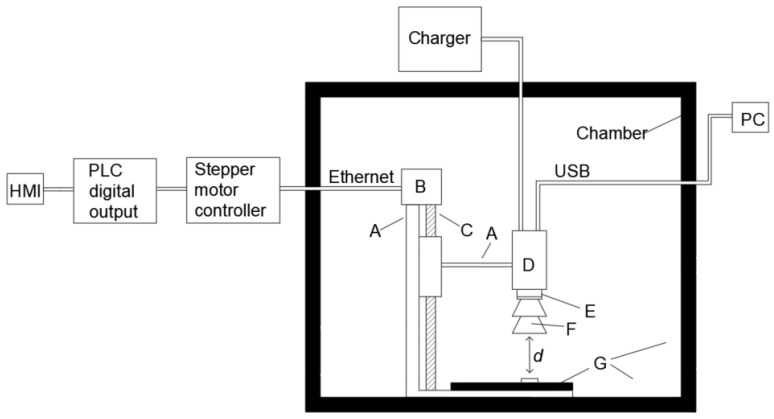
Thermographic camera and observed transistor placed in the prepared chamber. A—stand, B—stepper motor, C—screw, D—thermographic camera, E—thermographic camera lens, F—additional thermographic camera lens, G—polyurethane foam, d—distance between the tested transistor and the additional lens.

The distance *d* between the tested transistor and the additional lens was adjusted using a stepper motor. In turn, the stepper motor was controlled using a Siemens S7-1200 PLC controller (Siemens AG, Munich, Germany) [37]. A block diagram of the constructed stand is shown in Figure 3.

**Figure 4:**
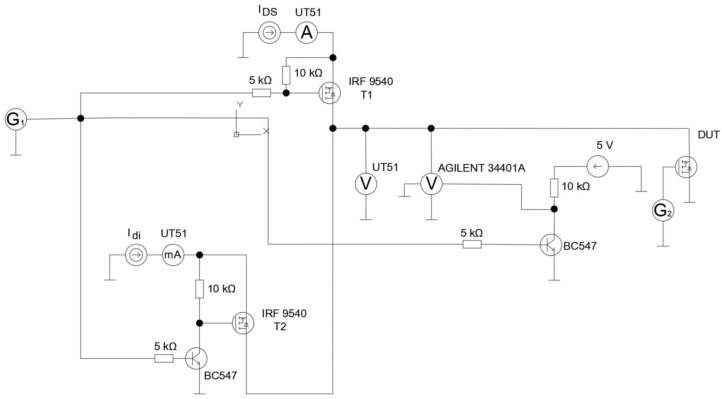
Diagram of the circuit enabling the evaluation of the influence of the switching frequency changes of the transistor on the temperature of its die. DUT—device under test, i.e., the tested transistor.

The observed transistor was connected to a circuit that allowed its switching frequency to be changed. The circuit diagram is shown in Figure 4. In this circuit, the transistor *T1* was turned on by the generator *G1* for 20 s. As a result, the load current *I_DS_* flowed through the tested transistor. During this time, the voltage drop between the drain and the source *V_DS_* was measured using an Agilent 34401A voltmeter. In the next step, the same generator *G1* turned on transistor *T2*, allowing the flow of the measuring current *I_di_* for 200 ms. This operation allowed estimating the die temperature based on the automatic measurement of the drop voltage *V_fd_* on the diode and the known characteristic between the drop voltage value and the die temperature. During the entire testing process, the tested transistor (DUT) was pulse-controlled using the *G2* generator with a PWM waveform with a duty cycle of 50% and a frequency in the range from 1 kHz to 50 kHz.

The case of the tested transistor, in which the switching frequency was changed, was observed using a thermographic camera. During the tests, first, for a given value of the current *I_DS_* flowing through the die and a given switching frequency *f_T_*, the temperature of the case (*T_c_*) was measured using a thermographic camera. We then waited until its value increased and stabilized at a specified level. When it was found that the *T_c_* value had stabilized, its thermographic measurement was performed. At the same time, *T_c_* was measured using a Pt1000 sensor, which was glued to the case near the thermographic measurement point (Figure 2). After the measurement was performed, the switching frequency *f_T_* of the transistor was changed for the same *I_D_* current value. The *f_T_* setting was changed for selected values, ranging from 1 kHz to 800 kHz.

### 2.2. Finite Element Analysis and Measurement of Die Temperature

The relationship between *T_c_* and *T_j_* was determined using finite element analysis (FEA), which is a numerical method used to solve problems in engineering and mathematical physics [38]. The software applied in the work performed was Solidworks 2020 SP05 (Dassault Systèmes, Vélizy-Villacoublay, France), which uses FEA, and the simulation was completed with the use of this software.

**Figure 5:**
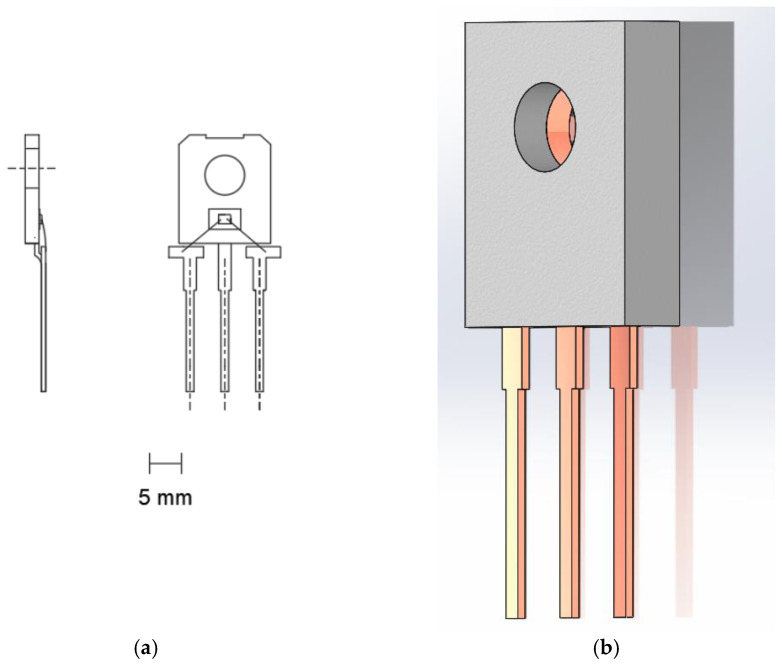
(**a**) Internal dimensions (see Figure 2 for details) and (**b**) three-dimensional model of the C2M0280120D transistor.

The simulation could be carried out after the transistor model had been constructed. Making the model required knowledge of its structure and internal dimensions. In order to determine these, the case of the tested transistor was opened and its interior was measured. For this purpose, a microscope equipped with a Cam 3.3 MP camera (Motic, Xiamen, China) was used. The microscope with the camera was calibrated using a special calibration glass. Based on the measurements taken, a three-dimensional model of the tested transistor was created. The model was created in Solidworks 2020 SP05 software. The created model and internal dimensions of the tested transistor C2M0280120D are shown in Figure 5.

After creating the model, all of its elements were assigned the material from which it was made, along with the thermal conductivity values *k*. Next, the simulation was started in the Solidworks 2020 SP05 environment. In the initial stage, we checked whether the temperature distribution (measured at the surface) changes after removing individual parts of the model (e.g., leads). The temperature distribution was also checked, depending on the given mesh size. After simplifying the model and selecting the mesh size, it was possible to determine the *T_j_* value based on the simulation work.

The die temperature (*T_j_*) of the tested transistors obtained as a result of simulation work was verified for the same conditions using the electrical method. In order to perform a reliable temperature measurement of the die using the electrical method, it was necessary to select the appropriate temperature-sensitive parameter (TSP). The voltage drop *V_fd_* across the body diode was chosen as the TSP. In order to use the TSP to determine the *T_j_* value, the relationship *T_j_* = *f*(*V_fd_*) had to be determined. For this reason, a measuring system was designed, the main element of which was a climatic chamber. The chamber used allowed for changing the temperature *T_a_* inside it. The *T_a_* value was changed in the range from 20 °C to 180 °C. Additionally, a Pt1000 sensor was placed inside the chamber, which was used to measure the temperature there. The sensor was connected in a four-wire circuit for measuring resistance using the technical method. A current of 100 µA flowed through the sensor.

**Figure 6:**
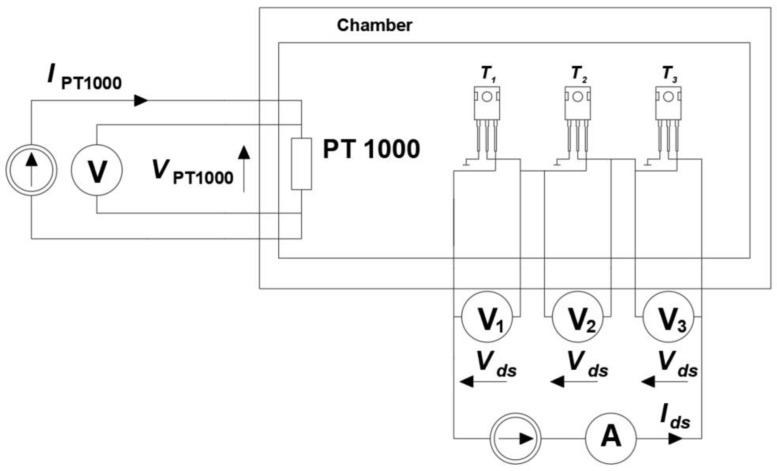
Measurement system enabling determination of the relationship *T_j_* = *f*(*V_fd_*).

In order to determine the relationship *T_j_* = *f*(*V_fd_*), three tested transistors were placed inside the described chamber. They were connected in such a way that the current *I_di_* (*I_di_* = 100 mA) forcing the voltage drop *V_fd_* on the body diode flowed through all diodes of the tested transistors (the body diodes of the three transistors were connected in series). The measurement setup is shown in Figure 6. The *V_fd_* values of all tested transistors were measured using an Agilent 34401A multimeter (Agilent, Santa Clara, CA, USA) [39]. The measurement was performed for a given temperature *T_a_* at the moment when the *V_fd_* voltage value stabilized. The constant values of the *V_fd_* voltage in time indicated that the temperature *T_a_* set in the chamber was equal to the die temperature *T_j_* of the transistors located in this chamber. In turn, the voltage drop *V*_Pt1000_ on the Pt1000 sensor was measured using a UT51 multimeter (UNI-T, Dongguan City, China) [40].

### 2.3. Power Dissipated in Die and Ambient Conditions

The correct simulation work using Solidworks 2020 SP05 requires determining the power *P* that has been released in the die and defining the boundary condition. The power released in the die can be determined using Equation (1):

(1) $$P=V_{DS}\cdot I_{DS}$$

where: *P*—power (in W) dissipated in the die, *V_DS_*—drop voltage (in V) between drain and source, *I_DS_*—current (in A) flowing between drain and source.

The *V_DS_* and *I_DS_* values were measured with measurement errors, which can be determined using the UT51 multimeter documentation (UNI-T, Dongguan City, China). Therefore, the *p* value will also be within the range defined by the measurement error limit ∆*P*, which can be determined from Equation (2):

(2) $$∆P=V_{DS}\cdot ∆{I_{DS}+I}_{DS}\cdot ∆V_{DS}$$

where: ∆*V_DS_*—limiting error of the *V_DS_* value (in V), ∆*I_DS_*—limiting error of the *I_DS_* value (in A). The ∆*I_DS_* and ∆*V_DS_* values can be determined using the formulas in the UT51 user manual [40].

The increase in die temperature is related to the distribution of effective power, *P_RMS_*, in the die. For this reason, the Equation (3) should be used:

(3) $$P_{RMS}=\sqrt{\frac{1}{T_{k}}\int_{t_{0}}^{t_{0}+T_{k}}P^{2}\left(t\right)dt}$$

where: *t*_0_—beginning of the period, *T_k_*—duration of the period.

The *P_RMS_* value is also within the range that is determined by the limiting error ∆*P_RMS_*_._ The limits of the range determined by ∆*P_RMS_* can be determined using Equation (2).

The temperature gradient in the radiative heat flux path between the transistor’s die and the transistor’s case can be determined using Equation (4):

(4) J=−k·∇·T

where: *J*—radiative heat flux (W∙m^−2^), ∇—Nabla operator.

Equation (4) can be written as Equation (5):

(5) $$J=-k\cdot \frac{dT}{dx}$$

where: *x*—distance between the points where the temperature values of the die and diode case were measured (m), *J*—radiative heat flux (W∙m^−2^).

In order to solve Equation (5), we need to separate the differentials that are on the right-hand side of the equation. Consequently, it is possible to integrate the equation on both sides. The constant of integration can be found using Equation (6):

(6) $$for\;x=0\to T=T_{1}\;for\;x=x_{k}\to T=T_{2}$$

where: *x_k_*—end point of the analyzed heat flow path (m), *T*_1_—temperature at the starting point of the analyzed heat flow path (K), *T*_2_—temperature at the end point of the analyzed heat flow path (K).

Consequently, it is possible to determine Equation (7):

(7) $$T_{1}-T_{2}=-\frac{P_{C}}{S\cdot k}\cdot x_{k}$$

where: *P_c_*—total power (in W) applied to the wall, *S*—area (m^2^) of the wall penetrated by *J* (W∙m^−2^).

Determining the correct temperature distribution in the transistor’s case (using Solidworks 2020 SP05 Software) requires determining the radiation coefficient *h_r_.* The *h_r_* coefficient defines the amount of thermal energy transferred to the environment by radiation per unit time, per unit area, and per unit temperature difference between the body radiating energy and the environment. The value of *h_r_* can be determined using Equation (8):

(8) $$h_{r}=\varepsilon \cdot \sigma \cdot (T_{S}\cdot T_{a})\cdot (T_{S}^{2}\cdot T_{a}^{2})$$

where: σ—Stefan–Boltzmann constant equal to 5.67 × 10^−8^ (W∙m^−2^∙K^−4^), *T_S_*—surface temperature (K), *T_a_*—air temperature (K).

It is also necessary to determine the value of the convection coefficient *h_cf_*, which defines the amount of thermal energy transferred to the environment by convection per unit time, per unit area, and per unit temperature difference between the body emitting the energy and the environment. To determine the *h_cf_* value for a flat surface, Equation (9) can be used:

(9) $$h_{cf}=\frac{Nu\cdot k}{L}$$

where: *h_cf_*—convection coefficient of flat surfaces, *Nu*—Nusselt number (-), *L*—characteristic length in meters (for a vertical wall, this value represents height).

The Nusselt number can be determined using Equation (10).

(10) $$Nu=a\cdot {\left(Pr\cdot Gr\right)}^{b}$$

where: *Gr*—Grashof number (-), *Pr*—Prandtl number (-), *a* and *b*—dimensionless coefficients. The values of coefficients *a* and *b* are provided in Table 1.

The Grashof number can be obtained from Equation (11):

(11) $$Gr=\frac{\alpha \cdot g\cdot (T_{s}-T_{a})\cdot \rho^{2}\cdot L^{3}}{\eta^{2}}$$

where: *g*—gravitational acceleration (9.8 m∙s^−2^), *α*—coefficient of expansion (0.0034 K^−1^), ρ—air density (1.21 kg∙m^−3^) at 273.15 K, *η*—dynamic air viscosity (1.75 × 10^−5^ kg∙m^−1^∙s^−1^) at 273.15 K.

Prandtl’s number is determined from Equation (12):

(12) $$Pr=\frac{c\cdot \eta }{k}$$

where: *Pr*—Prandtl’s number, *c*—specific heat of air (1005 J·kg^−1^·K^−1^) at 293.15 K.

When the value of the average linear velocity of the fluid flow is greater than 0 m/s, the Reynolds number must also be taken into account, which can be obtained using Equation (13):

(13) $$Re=\frac{V\cdot \rho \cdot L}{\eta }$$

where: *V*—average linear velocity of the fluid flow (m/s).

**Figure 7:**
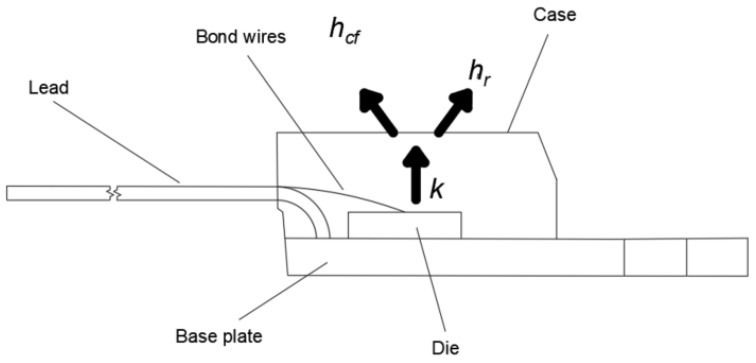
Analyzed heat flow path and its emission from the observed surface (by convection *h_cf_* and radiation *h_r_*). Thermal conductivity is designated as *k*.

In order to enable a better understanding of the boundary condition, the analyzed heat flow path and its emission by the observed surface (by convection *h_cf_* and radiation *h_r_*) are shown in Figure 7.

### 2.4. Uncertainties

The method by which the uncertainty of the thermographic temperature measurement *T_c_* can be determined is described in the document *Evaluation of the Uncertainty of Measurement in calibration* (EA-4/02 M: 2022) [41]. This is a method for determining the uncertainty of type B. In order to use this method, all input quantities *X_i_* that affect the result of the *T_c_* measurement and the range of their variability must be determined. This can be done based on experience and the literature. In this work, the thermographic camera processing equation from publication [42] was used (Equation (14)):

(14) $$T_{c}=\sqrt[{4}]{\frac{T_{cam}^{4}\cdot \varepsilon \cdot \sigma -\left(1-\varepsilon \right)\cdot \tau_{a}\cdot \sigma \cdot T_{refl}^{4}\cdot \tau_{l}-\left(1-\tau_{a}\right)\cdot \sigma_{c}\cdot T_{a}^{4}\cdot \tau_{l}-\left(1-\tau_{l}\right)\cdot \sigma_{c}\cdot T_{l}^{4}}{\varepsilon \cdot \tau_{a}\cdot \sigma_{c}\cdot \tau_{l}}}$$

where: *T_cam_*—temperature indicated by the thermographic camera without taking into account the influence of other factors, $\sigma_{c}$—Stefan-Boltzmann constant equal to 5.67 × 10^−8^ (W∙m^−2^∙K^−4^), *τ_a_*—atmosphere transmittance coefficient, *τ*_1_—transmittance of the thermographic camera lens, *T_a_*—air temperature, $T_{refl}$—reflected temperature, *T_l_*—thermographic camera lens temperature.

The next step is to determine the sensitivity coefficient *c_s_* for all input quantities from Equation (14). This is a derivative described in Equation (15):

(15) $$c_{s}(y)=\frac{\partial f}{\partial X_{i}}$$

where: *f_i_*—all input quantities from Equation (14).

In order to determine the uncertainty of the *T_c_* value, estimates of *x_i_* of the input quantities *X_i_* (for all above input quantities) must be determined. This is possible using Equation (16) (rectangular probability distribution):

(16) $$x_{i}=\frac{1}{2}(a_{+}+a_{-})$$

where: $a_{+}$—upper limit of the input quantity range, $a_{-}$—lower limit of the input quantity range.

Then, for each *X_i_*, the uncertainty standard *u*(*x_i_*) should be determined as per Equation (17):

(17) $${u^{2}(x}_{i})=\frac{1}{12}{\left(a_{+}-a_{-}\right)}^{2}$$

By multiplying the values of *u*(*x_i_*) and *c_s_*, we can obtain the uncertainty contribution *u*(*y*). The standard uncertainty *u*(*T_c_*) of the *T_c_* value can be obtained as the square root of the sum of squares of the values of *u*(*y*) as per Equation (18):

(18) $$u^{2}(T_{c})=\sum_{i=1}^{N}u(y)$$

In order to determine the expanded uncertainty *U*(*T_c_*), the value of *u*(*T_c_*) should be multiplied by the coverage factor *k*.

To determine ∆*T*_Pt1000_, Equations (19)–(23) can be used:

(19) $${\delta V}_{\mathrm{Pt}1000}=\frac{\Delta V_{\mathrm{Pt}1000}}{V_{\mathrm{Pt}1000}}\cdot 100$$

(20) $${\delta I}_{\mathrm{Pt}1000}=\frac{\Delta I_{\mathrm{Pt}1000}}{I_{\mathrm{Pt}1000}}\cdot 100$$

(21) $$\delta R_{\mathrm{Pt}1000}=\delta V_{\mathrm{Pt}1000}+\delta I_{\mathrm{Pt}1000}$$

(22) $$\Delta R_{\mathrm{Pt}1000}=\frac{R_{\mathrm{Pt}1000}\cdot \delta R_{\mathrm{Pt}1000}}{100}$$

where: $\Delta V_{Pt1000}$—limit error of *V*_Pt1000_, ${\delta V}_{Pt1000}$—relative error of *V*_Pt1000_, $\Delta I_{Pt1000}$—limit error of *I*_Pt1000,_ ${\delta I}_{Pt1000}$—relative error of *I*_Pt1000_, $\delta R_{Pt1000}$—relative error of *R*_Pt1000_, $\Delta R_{Pt1000}$—limit error of *R*_Pt1000_, *R*_Pt1000_—resistance of Pt1000 [42].

Then, by inserting the upper and lower range of Δ*R*_Pt1000_ into Equation (23), it is possible to obtain the upper and lower range of Δ*T*_Pt1000_ values:

(23) $$T_{\mathrm{Pt}1000}=10^{-5}\cdot R_{\mathrm{Pt}1000}^{2}+0.235\cdot R_{\mathrm{Pt}1000}-245.35+R_{\mathrm{Pt}1000}^{2}\cdot 4\cdot 10^{-7}\;-R_{\mathrm{Pt}1000}\cdot 2\cdot 10^{-5}+0.0011$$

To determine the Δ*V*_Pt1000_ and Δ*I*_Pt1000_ values, the documentation of the multimeter describe in reference [40] can be used.

## 3. Results

Using the measurement system shown in Figure 6, the relationship *T_jd_* = *f*(*V_fd_*) was determined. This relationship was approximated by the linear equation *y = e∙x + f*. As a result, the individual equations *T_jd_ = T_C_ + e∙V_fd_* + *f* were obtained for each transistor. The values of the coefficients for each transistor are given in Table 2.

**Figure 8:**
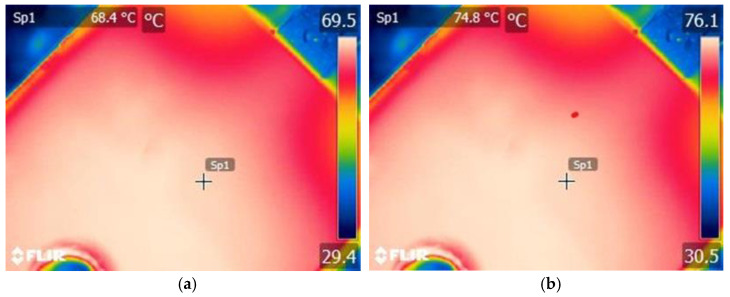
Examples of recorded thermograms: (**a**) *I_DS_* = 1 A; *f_T_* = 1 kHz, (**b**) *I_DS_* = 1 A; *f_T_* = 10 kHz. The thermograms were taken before gluing the Pt1000.

Then, each of the tested transistors was connected according to the diagram shown in Figure 4. The black part of the transistor case was observed with a thermographic camera. In order to minimize the factors disturbing the thermographic measurements, the observed transistors and the thermographic camera were placed in a chamber whose connection layout is shown in Figure 3. Additionally, *V_fd_* values were measured using a voltmeter. Using these values, the junction temperature *T_jd_* values were determined based on the previously determined relationship *T_jd_* = *f*(*V_fd_*) (Table 2). The *T_jd_* values determined in this way are given in Table 2, and sample recorded thermograms are shown in Figure 8.

In the next stage of the research, simulations were carried out using the FEM method. In the first step, a model of the tested transistor was designed. Materials and thermal conductivity values *k* are given in Table 3.

The selected values of the convection coefficients *h_cf_* of the observed surface (black part of the case) were in the range of 15.3 W/m^2^ K to 24.8 W/m^2^ K for the tested temperature ranges.

Additionally, the relationship between the mesh size *l* specified in the simulation parameters, the duration of a single simulation *t_s_*, and the accuracy of the determined temperature values D*T_S_* was checked. The obtained results are presented in Table 4.

**Figure 9:**
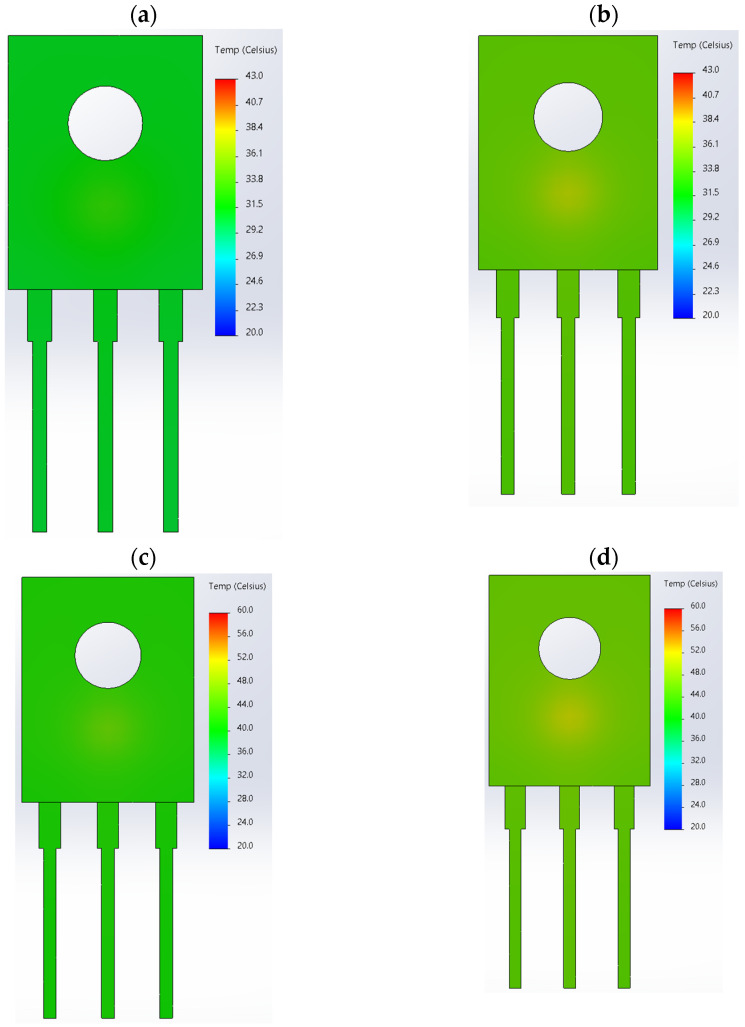

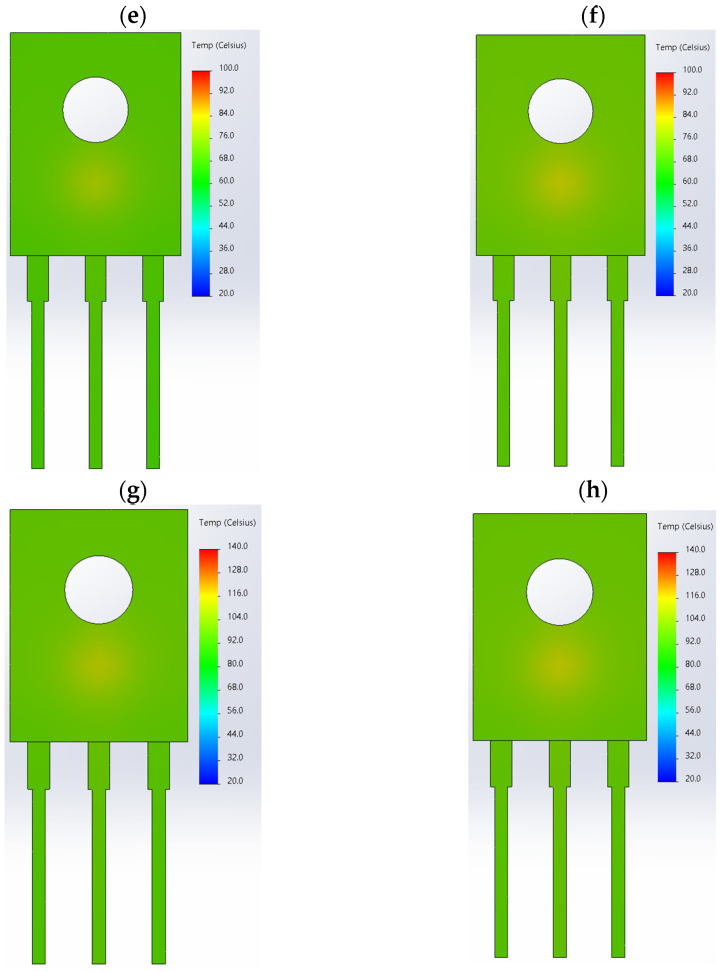
Examples of temperature distributions obtained from FEM simulations. (**a**) *I_DS_* = 0.25 A, *f_T_* = 1 kHz, (**b**) *I_DS_* = 0.25 A, *f_T_* = 500 kHz, (**c**) *I_DS_* = 0.5 A, *f_T_* = 1 kHz, (**d**) *I_DS_* = 0.5 A, *f_T_* = 500 kHz, (**e**) *I_DS_* = 1 A, *f_T_* = 1 kHz, (**f**) *I_DS_* = 1 A, *f_T_* = 500 kHz, (**g**) *I_DS_* = 1.5A, *f_T_* = 1 kHz, (**h**) *I_DS_* = 1.5A, *f_T_* = 500 kHz.

Based on the data presented in Table 4, a mesh size was selected at which the simulation duration was sufficiently short and the accuracy of the Δ*T_S_* temperature value obtained as a result of the simulation work was 0.1 °C (Table 4, No. 5). As a result, the *T_S_* temperature values obtained from the simulation work were close to the temperature *T_c_* recorded in the thermographic measurement for a given value of the power dissipated in the *P_RMS_* transistor. The selected mesh size was *l* = 1.0 mm. The example temperature distributions obtained from the simulation are shown in Figure 9.

Table 5, Table 6, Table 7 and Table 8 present the values of *T_c_* and *T_j_* recorded during measurements and the values of *T_S_* (transistor case) obtained as a result of simulation work, depending on the set value of the switching frequency *f_T_* of the transistor. The measurements were carried out for four current values.

In order to determine the uncertainty of the *T_c_* value, the range of all variables was determined from Equation (14). The adopted ranges of values and the determined *x_i_* are given in Table 9.

The *T_cam_* value was also taken into account. The *T_cam_* value limits were selected individually for each case (*T_cam_* ± 2 °C).

Then, using equations from Section 2.4, the standard uncertainty *u*(*x_i_*) and sensitivity coefficient *c_s_* (for all input quantities from Equation (14)) were determined. For each *X_i_*, the uncertainty contribution *u*(*y*) was determined. The *T_cam_* value was added to the budget with *c_s_* equal to 1. After constructing the uncertainty budget, the standard uncertainty *u*(*T_c_*) was determined. An example uncertainty budget for *f_t_* = 1 kHz and *I_DS_* = 0.25 A. *T_cc_* = 304.3 °C is shown in Table 10.

The value of *U*(*T_c_*) = 2.36 °C was obtained by multiplying the value of 1.18 by *k* = 2. Using the formulas presented in Section 2.4, the maximum value of ∆*T*_Pt1000_ of 1.73 °C was also determined.

## 4. Discussion

During the experimental work, an additional lens (Close-up 2×) was used with the thermographic camera. This enabled the thermographic camera used during the measurements (equipped with a 240 × 180 pixels detector matrix) to obtain such spatial resolution for which the edge of the field of view of a single detector was 67 µm. This value, taking into account the dimensions of the transistor shown in Figure 2, guaranteed that 25 fields of the view of a single detector of the thermographic camera (fields of the view placed in a rectangle of 5 × 5 pixels) were placed on the transistor case during the measurement. For this reason, the result of the thermographic temperature measurement can be considered reliable.

Before starting the measurements, the performance of the thermographic camera was compared with to the IRS Calilux radiation standard (Automation Technology, Bad Oldesloe, Germany). The results were compared in the range of 30–90 °C with a step of 5 °C. The largest difference between the standard and the camera was 0.72 °C (the camera error was ±2 °C or ±2%, whichever is greater). For this reason, the output from the thermographic camera can be considered reliable.

The results of the thermographic temperature measurements were comparable to those obtained using the Pt1000 sensor and to the results obtained during simulation work using the FEM method. During the work carried out, three transistor specimens were tested. Similar measurement results were obtained for each. Brand new Pt1000 sensors were used.

Analyzing the data from Table 5, Table 6, Table 7 and Table 8 (and especially comparing the die temperature (*T_j_*) determined based on the simulation and the voltage drop *T_jd_*) it can be seen that the largest difference was 4 °C. The conducted studies prove that the use of the transistor body diode during measurements allows for obtaining reliable results. They also prove that the results obtained by simulation work are confirmed in real conditions. Comparing the case temperature determined by simulation work (*T_S_*) with the temperature measured by means of a thermographic camera (*T_c_*), it can be seen that these values are the same. This proves that the model created is reliable.

Analyzing the data from Table 5, Table 6, Table 7 and Table 8, it can be seen that the difference between all results for *T*_*c*1_–*T*_*c*3_ are within the limit defined by the uncertainty *U*(*T_c_*). It can also be seen that the values of *T_c1_–T_c3_* and *T_S_* and *T*_Pt1000_ are within the range defined by ∆*T*_Pt1000_ and *U*(*T_c_*). For this reason, it can be assumed that the thermographic temperature measurement is reliable.

## 5. Conclusions

The aim of this research was to develop a method for performing indirect thermographic measurement of a SiC MOSFET and monitoring the SiC MOSFET temperature at variable switching frequencies.

Analyzing the transistor case temperatures measured with a thermographic camera (*T_c_*) at a frequency *f_t_*, it can be seen that despite the constant value of the *I_DS_* current, the *T_c_* value increases. The increase in the *T_c_* value depends on the *I_DS_* value. For the value of *I_DS_* = 0.25 A and *f_t_* in the range of 1 kHz–800 kHz, the *T_c_* value increased by 4.5 °C. For the value of *I_DS_* = 0.5 A and *f_t_* in the range of 1 kHz–800 kHz, the *T_c_* value increased by 5.6 °C. For the value of *I_DS_* = 1 A and *f_t_* in the range of 1 kHz–800 kHz, the *T_c_* value increased by 3 °C. For the value of *I_DS_* = 1.5 A and *f_t_* in the range of 1 kHz–800 kHz, the *T_c_* value increased by 1.5 °C. The *T_c_* value depends on the value of *I_DS_* and *f_t_*. With the increase in *I_DS_*, the *T_c_* value is set at increasingly lower values of *f_T_*.

The largest recorded difference between the case temperature and the die temperature was 27.3 °C. The use of a thermographic camera allows determining the temperature of the transistor die, which allows selecting the optimal control of the C2M0280120D transistor.

Due to the use of thermographic, there is no risk of electric shock as a result of touching the base plate or radiator, and the measurement result is obtained immediately. Based on a properly performed thermographic measurement of the temperature of the black part of the case (made of epoxy mold compound), it is possible to determine the temperature of the transistor die. As a result, its optimal operating point can be selected even more precisely. It is also possible to capture the operating point at which the transistor begins to operate incorrectly. This will prevent damage and save funds that would have to be spent in the event of a failure.

## Figures and Tables

**Table 1:** Values of coefficients *a* and *b* in Equation (10). lam—value for laminar flow, turb—value for turbulent flow.

Shape	Gr·Pr	*a_lam_*	*b_lam_*	*a_turb_*	*b_turb_*
Vertical flat wall	10^9^	0.59	0.25	0.129	0.33
Upper flat wall	10^8^	0.54	0.25	0.14	0.33
Lower flat wall	10^5^	0.25	0.25	NA	NA

**Table 2:** Values of the coefficients *e* and *f* of the curves *T_jd_ = T_C_ + e∙V_fd_ + f* of the tested transistors.

No	Transistor	*e*	*f*
1	C2M0280120D	8.9582	1.6507
2	C2M0280120D	8.9684	1.6012
3	C2M0280120D	8.9549	1.6577

**Table 3:** Materials specified in the 3D model and their thermal conductivity values *k*.

Internal Structure Component	Material	*k* [W/m∙K]
Black part of the case	EME 590	0.25
Back part of the case	Copper	400
Semiconductor element	Silicon carbide	150
Left lead	Copper	400
Internal lead	Copper	400
Right lead	Copper	400
Internal connections	Copper	400
Grease	Melamine resin	0.20

**Table 4:** Mesh size *l*, simulation duration *t_s_*, and temperature values *T_S_* obtained during FEM simulation defined in the simulation parameters.

No.	*t_s_* [s]	*l* [mm]	Δ*T_S_* [°C]
1	3	4.0	1.9
2	5	3.0	1.4
3	8	2.0	0.6
4	15	1.5	0.4
5	41	1.0	0.1
6	499	0.5	0.1

**Table 5:** Transistor case temperature *T_c_* determined from thermographic measurements, junction temperature *T_jd_* determined from the relationship *T_jd_* = *f*(*V_fd_*), and transistor case temperature *T_S_* and junction temperature *T_j_* determined from simulations, depending on the set switching frequency *f_T_*. The results were obtained for the current *I_DSmax_* = 0.25 A. *T*_*c*1_ is the *T_c_* value measured for the first transistor, *T*_*c*2_ is the *T_c_* value measured for the second transistor, and *T*_*c*3_ is the *T_c_* value measured for the third transistor.

No.	*f_T_*	*I_DS_*	*P*	*T_c_*_1_	*T_c_*_2_	*T_c_*_3_	*T_jd_*	*T_S_*	*T_j_*	*T*_Pt1000_
	[kHz]	[A]	[W]	[°C]	[°C]	[°C]	[°C]	[°C]	[°C]	[°C]
1	1	0.25	0.52	33.1	33.3	32.6	53.4	33.0	49.4	31.1
2	5	0.25	0.52	33.4	33.6	32.9	53.7	33.3	49.8	31.4
3	10	0.25	0.52	33.6	33.8	33.1	53.9	33.5	50.1	31.6
4	50	0.25	0.52	33.9	34.2	33.3	54.2	33.8	50.5	31.9
5	100	0.25	0.52	34.3	34.6	33.7	54.6	34.2	51.1	32.3
6	150	0.25	0.52	35.0	35.3	34.4	55.3	34.9	52.1	32.9
7	200	0.25	0.52	35.6	35.9	35.0	55.9	35.5	53.1	33.6
8	300	0.25	0.52	36.6	36.9	36.0	56.9	36.5	54.2	34.6
9	400	0.25	0.52	36.9	37.2	36.3	57.2	36.8	54.9	34.9
10	500	0.25	0.52	37.3	37.6	36.7	57.5	37.2	55.5	35.3
11	600	0.25	0.52	37.1	37.4	36.5	57.3	37.0	55.1	35.1
12	700	0.25	0.52	37.3	37.6	36.7	57.5	37.2	55.3	35.3
13	800	0.25	0.52	37.6	37.9	37.0	57.8	37.5	55.7	35.6

**Table 6:** Transistor case temperature *T_c_* determined from the thermographic measurements, junction temperature *T_jd_* determined from the relationship *T_jd_* = *f*(*V_fd_*), and the transistor case temperature *T_S_* and junction temperature *T_j_* determined from simulations, depending on the set switching frequency *f_T_*. The results were obtained for the current *I_DSmax_* = 0.5 A. *T*_*c*1_ is the *T_c_* value measured for the first transistor, *T*_*c*2_ is the *T_c_* value measured for the second transistor, *T*_*c*3_ is the *T_c_* value measured for the third transistor.

No.	*f_T_*	*I_DS_*	*P*	*T*_*c*1_	*T*_*c*2_	*T*_*c*3_	*T_jd_*	*T_S_*	*T_j_*	*T*_Pt1000_
	[kHz]	[A]	[W]	[°C]	[°C]	[°C]	[°C]	[°C]	[°C]	[°C]
1	1	0.5	1.20	45.7	46.1	45.3	68.8	45.7	66.3	43.7
2	5	0.5	1.19	45.7	46.1	45.3	68.7	45.7	66.3	43.7
3	10	0.5	1.19	46.0	46.4	45.6	69.0	46.0	66.7	44.0
4	50	0.5	1.18	47.0	47.4	46.6	69.8	47.0	67.2	45.0
5	100	0.5	1.18	48.1	48.5	47.7	70.8	48.1	68.7	46.1
6	150	0.5	1.17	49.2	49.6	48.8	71.8	49.2	69.3	47.2
7	200	0.5	1.17	50.1	50.5	49.7	72.7	50.1	70.7	48.0
8	300	0.5	1.17	50.9	51.3	50.5	73.5	50.9	71.9	48.8
9	400	0.5	1.17	50.7	51.1	50.3	73.3	50.7	71.6	48.6
10	500	0.5	1.17	51.0	51.4	50.6	73.5	51.0	71.1	48.9
11	600	0.5	1.17	51.6	52.0	51.2	74.1	51.6	72.0	49.5
12	700	0.5	1.17	51.5	51.9	51.1	74.0	51.5	71.8	49.4
13	800	0.5	1.16	51.3	51.7	50.9	73.8	51.3	71.5	49.2

**Table 7:** Transistor case temperature *T_c_* determined from thermographic measurements, junction temperature *T_jd_* determined from the relationship *T_jd_* = *f*(*V_fd_*), and transistor case temperature *T_S_* and junction temperature *T_j_* determined from simulations, depending on the set switching frequency *f_T_*. The results were obtained for the current *I_DSmax_* = 1 A. *T*_*c*1_ is the Tc value measured for the first transistor, *T*_*c*2_ is the Tc value measured for the second transistor, *T*_*c*3_ is the *T_c_* value measured for the third transistor.

No.	*f_T_*	*I_DS_*	*P*	*T*_*c*1_	*T*_*c*2_	*T*_*c*3_	*T_jd_*	*T_S_*	*T_j_*	*T*_Pt1000_
	[kHz]	[A]	[W]	[°C]	[°C]	[°C]	[°C]	[°C]	[°C]	[°C]
1	1	1	2.90	80.0	80.6	79.7	107.7	80.1	106.1	77.8
2	5	1	2.90	80.4	81.0	80.1	108.1	80.5	106.3	78.2
3	10	1	2.89	79.9	80.5	79.6	107.6	80.0	106.1	77.7
4	50	1	2.88	81.6	82.2	81.3	109.1	81.7	106.6	79.4
5	100	1	2.87	81.9	82.5	81.6	109.4	82.0	106.8	79.7
6	150	1	2.86	81.7	82.3	81.4	109.1	81.8	106.5	79.5
7	200	1	2.86	82.4	83.0	82.1	109.8	82.5	106.8	80.1
8	300	1	2.85	83.0	83.6	82.7	110.3	83.1	106.8	80.7
9	400	1	2.85	83.1	83.7	82.8	110.4	83.2	106.9	80.8
10	500	1	2.85	83.1	83.7	82.8	110.4	83.2	106.9	80.8
11	600	1	2.85	82.9	83.5	82.6	110.1	83.0	106.7	80.6
12	700	1	2.85	83.1	83.7	82.8	110.3	83.2	106.7	80.8
13	800	1	2.85	83.0	83.6	82.7	110.2	83.1	106.6	80.7

**Table 8:** Transistor case temperature *T_c_* determined from thermographic measurements, junction temperature *T_jd_* determined from the relationship *T_jd_* = *f*(*V_fd_*), and transistor case temperature *T_S_* and junction temperature *T_j_* determined from simulations, depending on the set switching frequency *f_T_*. The results were obtained for the current *I_DSmax_* = 1.5 A. *T*_*c*1_ is the *T_c_* value measured for the first transistor, *T*_*c*2_ is the *T_c_* value measured for the second transistor, *T*_*c*3_ is the *T_c_* value measured for the third transistor.

No.	*f_T_*	*I_DS_*	*P*	*T*_*c*1_	*T*_*c*2_	*T*_*c*3_	*T_jd_*	*T_S_*	*T_j_*	*T*_Pt1000_
	[kHz]	[A]	[W]	[°C]	[°C]	[°C]	[°C]	[°C]	[°C]	[°C]
1	1	1.5	3.30	111.9	112.6	111.8	141.9	112.1	140.0	109.5
2	5	1.5	3.29	113.3	114.0	113.2	143.2	113.5	140.7	110.8
3	10	1.5	3.28	113.4	114.1	113.3	143.2	113.6	140.9	110.9
4	50	1.5	3.27	113.2	113.9	113.1	142.9	113.4	140.5	110.7
5	100	1.5	3.26	113.3	114.0	113.2	142.9	113.5	140.7	110.8
6	150	1.5	3.25	113.4	114.1	113.3	143.0	113.6	140.9	110.9
7	200	1.5	3.24	113.3	114.0	113.2	142.8	113.5	140.7	110.8
8	300	1.5	3.22	113.4	114.1	113.3	142.7	113.6	140.9	110.9
9	400	1.5	3.22	113.4	114.1	113.3	142.7	113.6	140.9	110.9
10	500	1.5	3.22	113.4	114.1	113.3	142.7	113.6	140.9	110.9
11	600	1.5	3.22	113.3	114.0	113.2	142.6	113.5	140.7	110.8
12	700	1.5	3.22	113.4	114.1	113.3	142.7	113.6	140.9	110.9
13	800	1.5	3.22	113.4	114.1	113.3	142.7	113.6	140.9	110.9

**Table 9:** Values of the variables from Equation (14) and the determined *x_i_*.

No	Value	Unit	*a_+_*	*a_−_*	*x_i_*
1	*ε*	-	0.98	0.96	0.97
2	*τ* *_a_*	-	1.00	0.98	0.99
3	*T_refl_*	°C	5.00	0.00	2.50
4	*τ* *_l_*	-	1.00	0.98	0.99
5	*T_a_*	°C	30.00	16.00	22.5
6	*T_l_*	°C	30.00	16.00	22.5

**Table 10:** Example uncertainty budget for *f_t_* = 1 kHz and *I_DS_* = 1.5 A *T*_*c*1_ = 112.1 °C.

Symbol	Unit	*x_i_*	*u*(*x_i_*)	Probability Distribution	*c_s_*	*u_i_*(*y*)
*τ_a_*	-	0.99	0.01	normal	0.45	0.01
*ε*	-	0.95	0.03	rectangular	−7.65	−0.22
*T_refl_*	°C	20.85	1.44	rectangular	0.01	0.02
*τ_l_*	-	0.99	0.01	rectangular	−10.32	−0.06
*T_a_*	°C	20.85	4.04	rectangular	−0.02	−0.06
*T_l_*	°C	20.85	4.04	rectangular	−0.02	−0.06
*T_cam_*	°C	20.85	1.15	rectangular	1.00	1.15
*T_c_*	°C	112.1				1.18

## Data Availability

Data are contained within the article.

## Nomenclature

*a* and *b*	dimensionless coefficients
$a_{+}$	upper limit of the input quantity range
$a_{-}$	lower limit of the input quantity range
*a_lam_*	value of coefficient *a* for laminar flow
*b_lam_*	value of coefficient *b* for laminar flow
*a_turb_*	value of coefficient *a* for turbulent flow
*b_turb_*	value of coefficient *b* for turbulent flow
*c*	specific heat of air equal to 1005 J·kg^−1^·K^−1^ at 293.15 K
*c_s_*	sensitivity coefficient
*d*	distance between the tested transistor and the additional lens
DUT	device under test
*e*	slope of the equation *T_jd_* = f(*V_fd_*) of the transistor
*f*	free term of the equation *T_jd_* = f(*V_fd_*) of the transistor
FEM	finite element method
*f_i_*	all input quantities from Equation (14)
*f_T_*	switching frequency
*g*	gravitational acceleration (9.8 m∙s^−2^)
*Gr*	Grashof number
*h_cf_*	convection coefficient of flat surfaces
*h_r_*	radiation coefficient
*I_D_*	drain current
*I_DS_*	drain—source current (in A)
*I*_Dpulse_	pulse current
*I*_Pt1000_	current flowing through Pt1000
IFOV	instantaneous field of view
IR	infrared radiation
*J*	radiative heat flux
*K*	thermal conductivity
*k*	coverage factor
*L*	characteristic length in meters (for a vertical wall, this value represents height)
*Nu*	Nusselt number
*P_RMS_*	power supplied to the transistor
*P*	power dissipated in the die (in W)
*P_c_*	total power applied to the wall
*Pr*	Prandtl number
*Re*	Reynolds number
*R*_Pt1000_	resistance of Pt1000
*S*	area of the wall penetrated by *J*
*T_a_*	ambient temperature
*T_c_*	case temperature
*T*_*c*1_	*T_c_* value measured for the first transistor
*T*_*c*2_	*T_c_* value measured for the second transistor
*T*_*c*3_	*T_c_* value measured for the third transistor
*T_cam_*	temperature indicated by the thermographic camera
*T_k_*	duration of the period
*T_j_*	junction temperature
*T_d_*	junction temperature determined electrically
*T_l_*	thermographic camera lens temperature
*T*_Pt1000_	temperature value measured with Pt1000
$T_{refl}$	reflected temperature
*T*_1_	temperature at the starting point of the analyzed heat flow path (K)
*T*_2_	temperature at the end point of the analyzed heat flow path (K)
*t*_0_	beginning of the period
*u*(*x_i_*)	standard uncertainty of each *X_i_,*
*u*(*y*)	uncertainty contribution
*u*(*T_c_*)	standard uncertainty of *T_c_* values
*U*(*T_c_*)	expanded uncertainty of *T_c_* values
*V*	average linear velocity of the fluid flow
*V_DS_*	drop voltage between drain and source (in V)
*V_Dsmax_*	maximum drain—source voltage
*V_fd_*	diode forward voltage
*V_GS_*	gate—source voltage
*V_GSmax_*	maximum gate—source voltage
*V*_Pt1000_	voltage drop on Pt1000
*x*	distance between the points where the temperature values of the die and diode case were measured, and J is the radiative heat flux
*x_k_*	end point of the analyzed heat flow path
*X_i_*	input quantities
*x_i_*	estimates of the input quantities *X_i_*
∇	Nabla operator
*α*	coefficient of expansion equal to 0.0034 K^−1^
*ε*	emissivity factor
∆*I_DS_*	limiting error of the *I_DS_* value (in A)
∆*V_DS_*	limiting error of the *V_DS_* value (in V)
$\Delta I_{\mathrm{Pt}1000}$	limit error of *I*_Pt1000_
${\delta I}_{\mathrm{Pt}1000}$	relative error of *I*_Pt1000_
∆*P*	limiting error of the *P* value in W
$\delta R_{\mathrm{Pt}1000}$	relative error of *R*_Pt1000_
$\Delta R_{\mathrm{Pt}1000}$	limit error of *R*_Pt1000_
∆*T*_Pt1000_	limit error of ∆*T*_Pt1000_ of measurement *T*_Pt1000_
$\Delta V_{\mathrm{Pt}1000}$	limit error of *V*_Pt1000_
${\delta V}_{\mathrm{Pt}1000}$	relative error of *V*_Pt1000_
η	dynamic air viscosity equal to 1.75 × 10^−5^ kg∙m^−1^∙s^−1^ at 273.15 K
ρ	density equal to 1.21 (kg∙m^−3^) in 273.15 (K)
σ_c_	Stefan–Boltzmann constant
*τ_a_*	atmosphere transmittance coefficient
*τ_l_*	transmittance of the thermographic camera lens
